# Supplementary material for: Integrated modeling of the Nexin-dynein regulatory complex reveals its regulatory mechanism
Source: Nat Commun. 2023 Sep 15;14:5741. doi: 10.1038/s41467-023-41480-7 (PMC10504270; doi:10.1038/s41467-023-41480-7)
Supplement: Supplementary file 1 — Supplementary Information [file 41467_2023_41480_MOESM1_ESM.pdf]

Supplementary Information

Ghanaeian et al. Integrated modeling of the Nexin-dynein regulatory complex reveals its regulatory mechanism. *Nat Commun*, 2023

Table of content

Supplementary Figures 2

Supplementary Tables...11

Supplementary Movies 20

References...21

Supplementary Figures

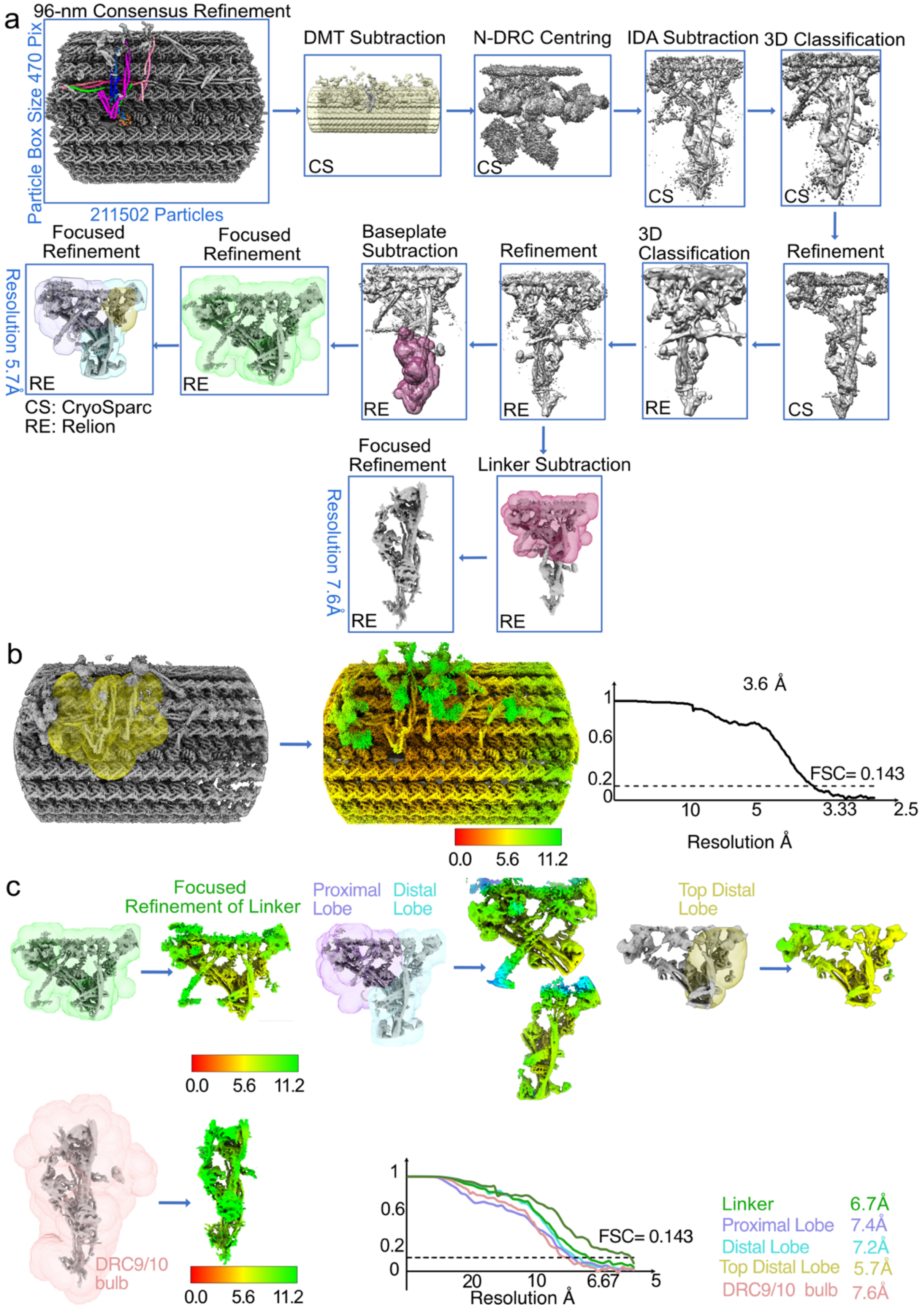

Supplementary Figure 1. The workflow for reconstruction of the base plate and linker regions of the N-DRC. a The overall workflow for reconstruction of the N-DRC. DMT signal was subtracted, and N-DRC was centered. After refinement, the IDA signal was subtracted. In the next step, the N-DRC structure underwent several 3D classifications and refinements in Cryosparc (CS) and Relion (RE). To resolve the linker domain, the baseplate signal was subtracted, and a consensus refinement was performed using a mask covering the entire linker domain. Finally, focused refinement was conducted on the distal and proximal lobes of the N-DRC. To resolve the bulb of the DRC9/10 structure, the linker domain signal was subtracted, and the remaining structure underwent focused refinement. **b** Refinement of the base plate region. The mask is shown as a transparent density (left panel). The resulting map is shown in the middle panel. The Fourier Shell Correlation of the baseplate domain of the N-DRC cryo-EM map shows 3.6 Å resolution (right panel). **c** Refinement of the linker region. The linker region resolved at 6.7 Å. The distal and proximal lobes have 7.2 Å and 7.4 Å resolution, respectively. The top distal lobe resolved at 5.7 Å. Last, the bulb of the DRC9/10 resolved at 7.6 Å.

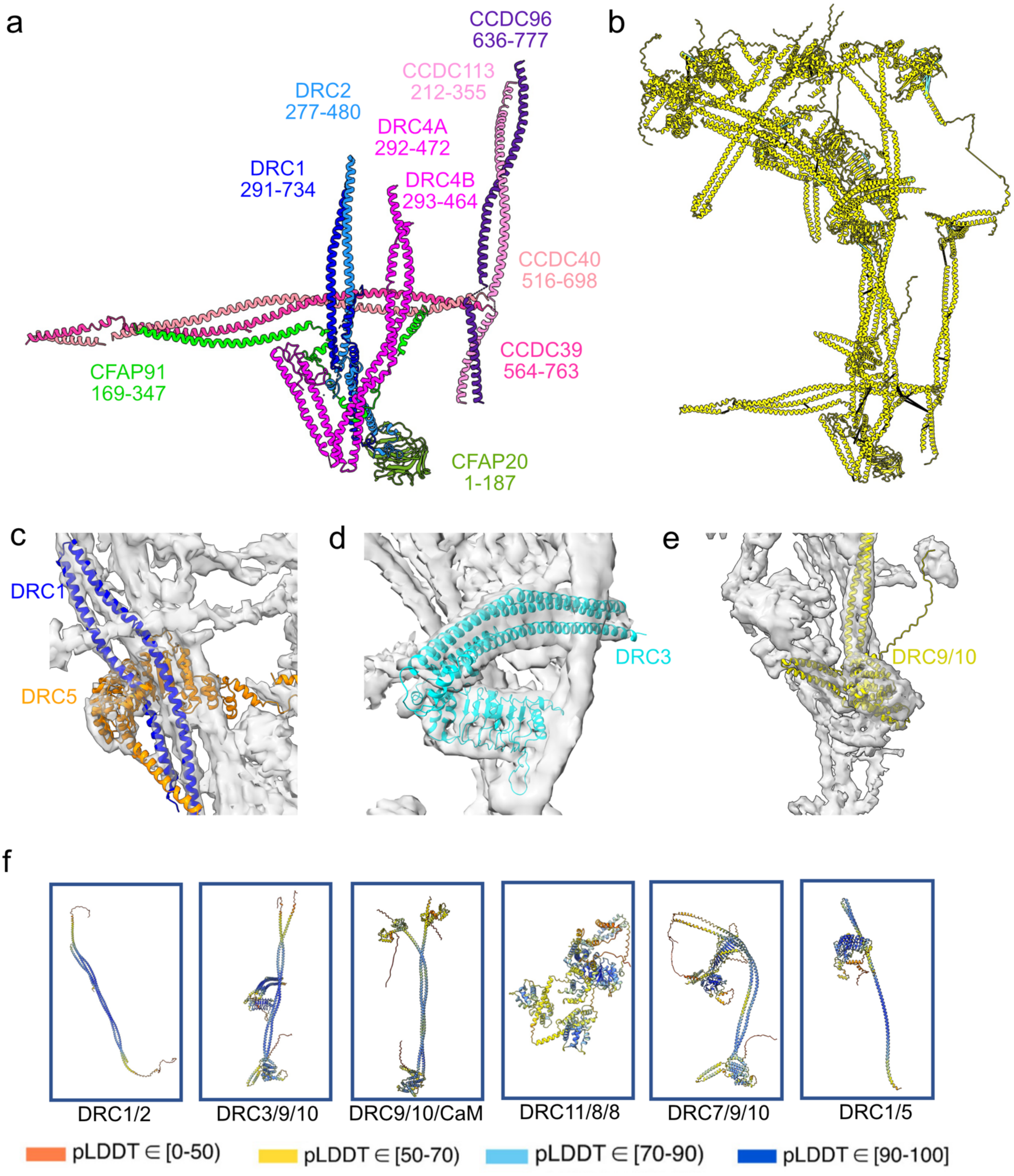

Supplementary Figure 2. AlphaFold2 modelling of all N-DRC components. a The protein parts in the baseplate part were modelled using Coot. **b** The intra-molecular (cyan lines) and inter-molecular (black lines) cross-links are identified in the N-DRC. **c** Fitting of DRC1 and DRC5 in the linker map. **d** DRC3 was fitted as a bundle of coiled-coil structures in the N-DRC linker map. **e** The DRC9/10 N-terminus is fitted to the N-DRC map. **f** The AlphaFold2 Multimer models of the DRC proteins and CCDC96/113 are coloured based on the pLDDT score where blue shows the highest score, and orange represents the lowest score.

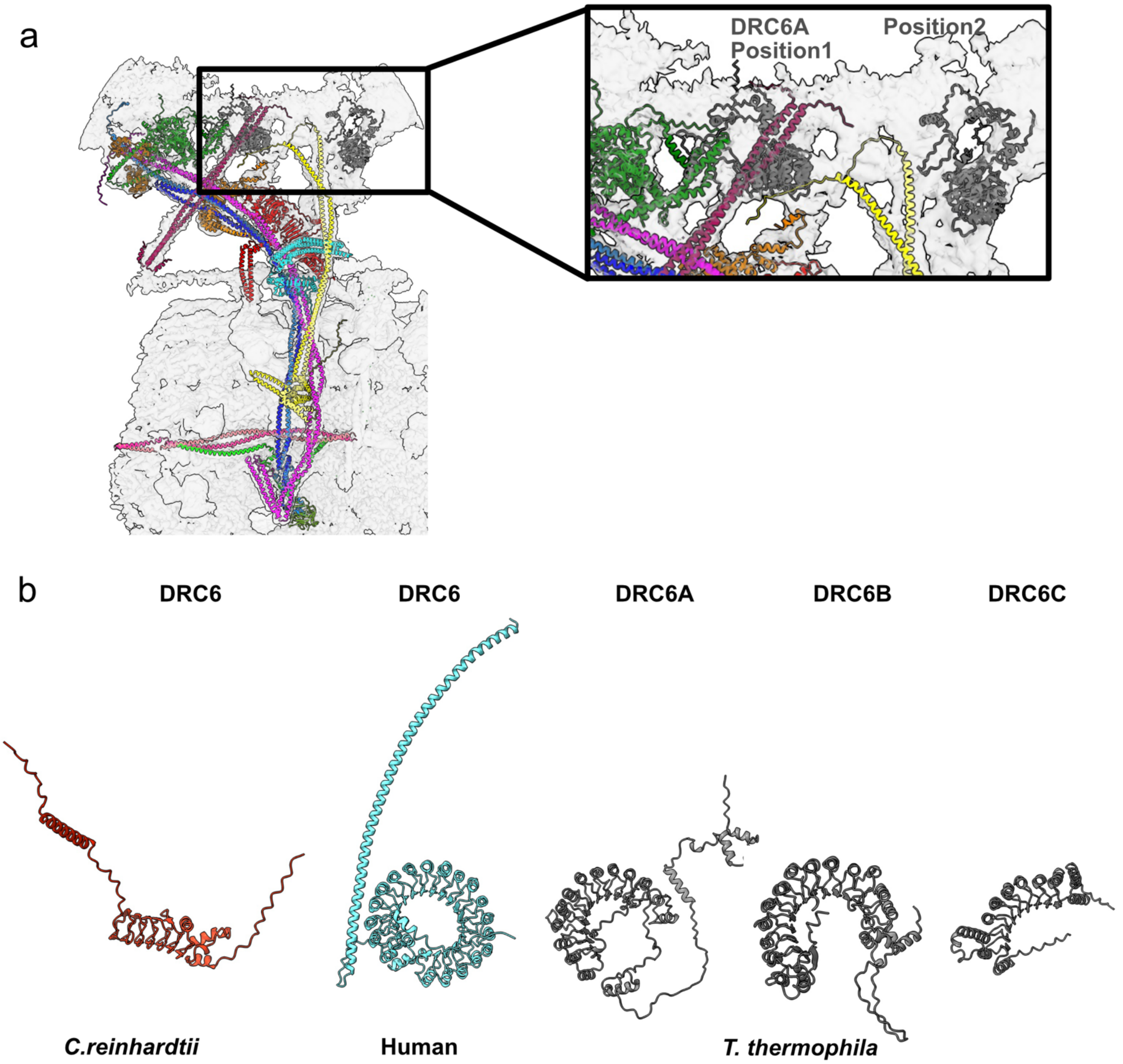

Supplementary Figure 3. Localizing DRC6A in the N-DRC. a Two potential locations for DRC6A in the cryo-EM map identified by integrated modelling. **b** The structure of DRC6 in three different species. There are three paralogs of DRC6 (A/B/C) in *T. thermophila*.

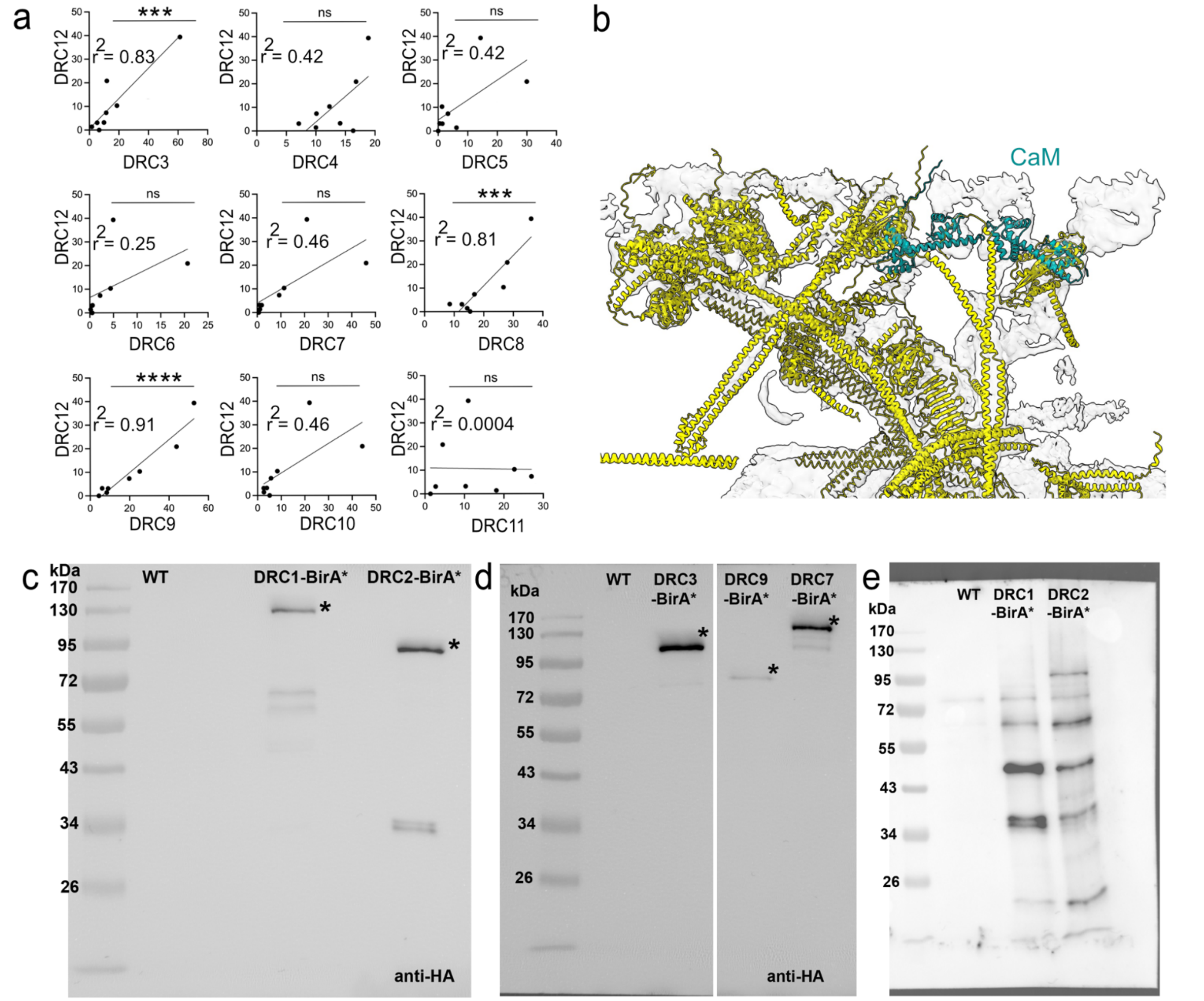

Supplementary Figure 4. Identification of new N-DRC components. a Correlation graphs of consensus normalized RNA expression levels for DRC components vs. the newly identified DRC12. The coefficient of determination (R2) of the linear regression is indicated. *$p < 0.05$; ***$p < 0.001$;****$p < 0.0001$; ns: Not Significant. **b** The approximate localization of calmodulin in the N-DRC structure. **c-e** Western blots with HA antibody of purified cilia from *WT*, *DRC1-HA-BirA** and *DRC2-HA-BirA** cells **(c)** and *DRC3-HA-BirA*, DRC7-HA-BirA** and *DRC9-HA-BirA** **(d)** indicate the baits are in the cilia. **e** Western blots using Streptavidin-HRP of purified cilia from *WT, DRC1-HA-BirA** and *DRC2-HA-BirA** cells after 4 h incubation with 50 μM biotin at 30°C showed that biotinylation of ciliary components happens. The Western blot experiments were conducted with a rigorous approach to ensure the reliability of the results. Each experiment was independently repeated a minimum of two to three times.

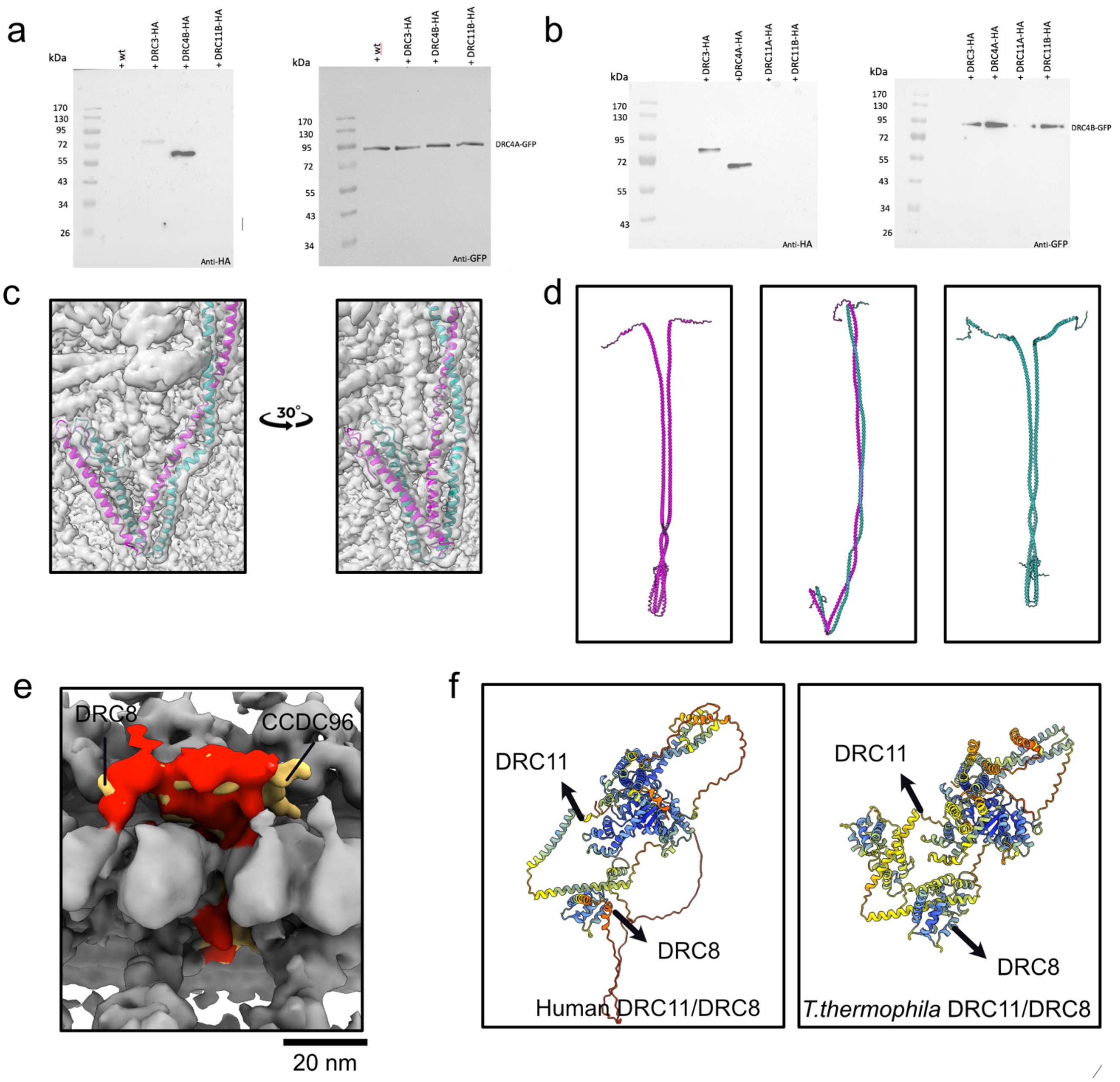

Supplementary Figure 5. The interaction of DRC4 paralogs in the N-DRC. a-b Western blots using HA and GFP antibodies showed proteins pulled down by (A) DRC4A-GFP or (B) DRC4B-GFP bound to anti-GFP beads. The pull-down assays indicate that DRC4A interacts with DRC4B. DRC4B seems to interact strongly with DRC3 compared to DRC4A. Each Western blot experiment was repeated three times to ensure the reproducibility and validity of the results. **c** The green and magenta colours show the parts of DRC4A and DRC4B modelled by Coot in the base plate, respectively. **d** The AlphaFold2 Multimer model of DRC4B/DRC4B, DRC4A/DRC4B, and DRC4A/DRC4A. The prediction of DRC4A/DRC4B complex is the most similar to the structure observed in the base plate region. These two proteins are different in sequence with an identity score of 25.39. **e** Superimposed subtomogram averaged map of the DMT from human (EMD-5950) (red) and *T. thermophila* (yellow). The larger density in the proximal and distal lobes of the *T. thermophila* map indicates the second copy of DRC8 and N-terminus of CCDC96, respectively, because the human map lacks those densities. Scale bar, 20 nm. **f** The AlphaFold2 Multimer prediction of DRC11/DRC8 subcomplex for humans and *T. thermophila*. The AlphaFold2 prediction indicates only one copy of DRC8 binds to DRC11 in humans while two copies of DRC8 bind to DRC11 in *T. thermophila*. The AlphaFold2 models are coloured based on the pLDDT score in which blue represents the highest confidence and orange represents the lowest confidence.

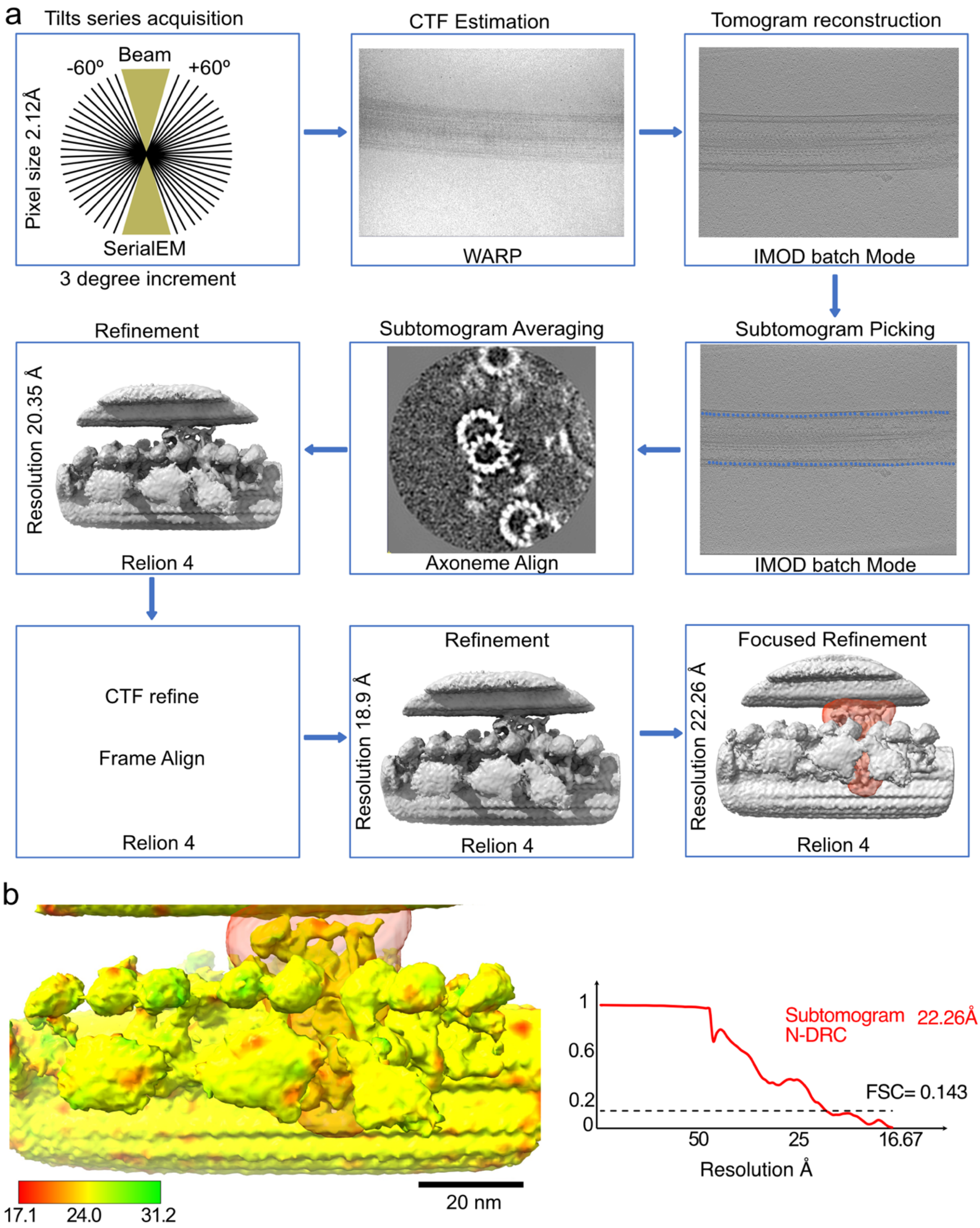

Supplementary Figure 6. The workflow of cryo-ET and subtomogram averaging of the WT cilia. a Tilt series were acquired using dose-symmetric scheme ranging from -60 to 60 degrees in 3-degree increment. Then, CTF estimation of the tilt series was performed in WARP. The tomogram reconstruction was done using IMOD batch mode. Then, subtomogram picking and subtomogram averaging for the 96nm repeat of the DMT were done using IMOD and Axoneme Align software. The alignment parameters were then converted into Relion 4 star files for refinement. Within Relion 4, CTF refinement and frame alignment were done before the final 96-nm DMT refinement and focused refinement of the N-DRC region. **b** The local resolution estimation of the subtomogram average of the N-DRC. The Fourier Shell Correlation shows a global resolution of 22.26 Å of the subtomogram average. Scale bar, 20 nm.

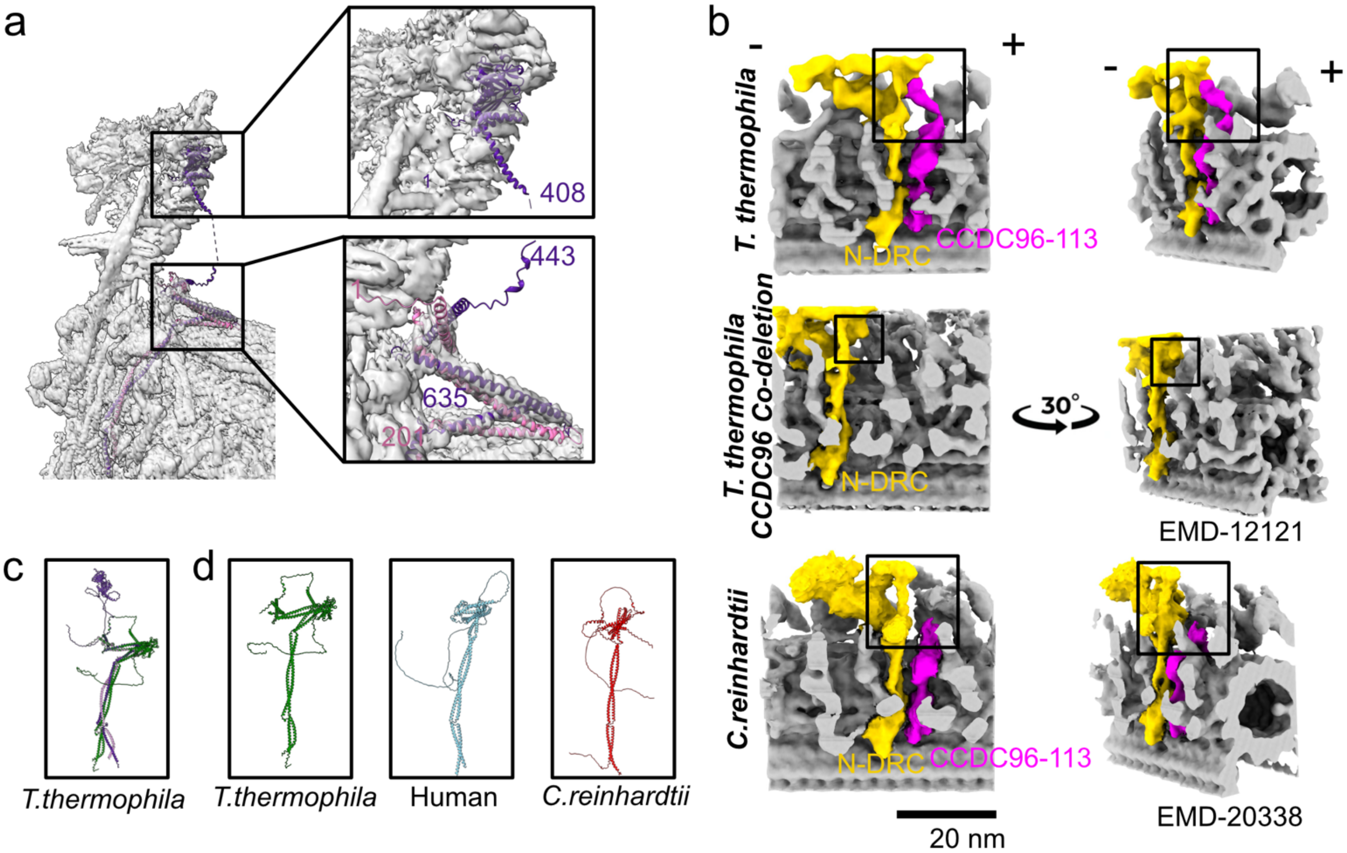

Supplementary Figure 7. The structure of the CCDC96/113 complex. a Fitting the N-terminal domain of CCDC96 in the cryo-EM map of the linker region. The numbers represent the amino acid number in the CCDC96 sequence. **b** The cryo-ET maps of the 96-nm DMT from *T. thermophila* wild-type, *T. thermophila* CCDC96 Co-Deletion (EMD-12121), and *C. reinhardtii* (EMD-20338) show that only in the *T. thermophila* WT map, there is a density connecting the CCDC96/113 complex (purple) to the N-DRC (yellow). This density is likely to correspond to the non-conserved N-terminus of CCDC96 in *T. thermophila*. The IDAs are deleted for better visualization. Signs (+) and (-) indicate the distal and proximal ends of the DMT. **c** AlphaFold2 multimer prediction of CCDC96/113 complexes from *T. thermophila*. Green colour indicates AphaFold2 multimer structure before truncation and refinement. The purple colour shows protein after truncation and refinement. **d** AlphaFold2 multimer prediction of CCDC96/113 complexes from *T. thermophila*, human and *C. reinhardtii* show that *T. thermophila* CCDC96 has an extra N-terminal globular domain. Scale bar, 20 nm.

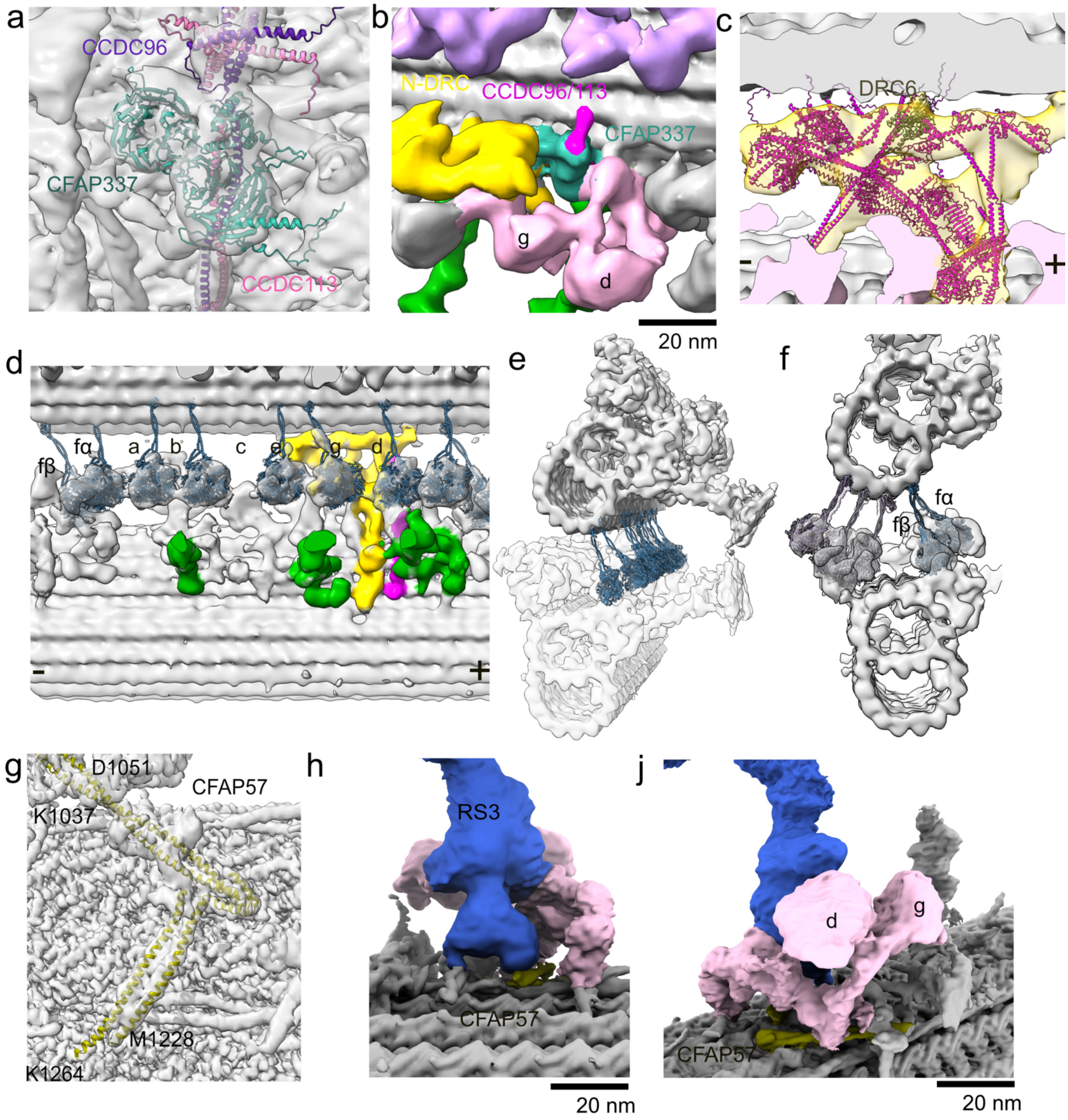

Supplementary Figure 8. Identifications and interactions of N-DRC associated proteins. **a** Fitting the AlphaFold2 predicted structure of CFAP337 in the cryo-EM map showing potential interactions with CCDC96/113. **b** The subtomogram average shows that CFAP337 (turquoise) interacts with CCDC96/113 (purple) and stems of dyneins g and d (light pink). The N-DRC is in yellow. Scale bar, 20 nm. **c** Fitting of the N-DRC pseudo-atomic model in the subtomogram average showing the potential interactions of the flexible loops with the adjacent DMT. **d** Fitting of dynein heavy chains into the IDA densities in the subtomogram average. Colors: N-DRC, yellow; RS, green; CCDC96/113, purple. Signs (+) and (-) indicate the distal and proximal ends of the DMT. **e** The stalks of IDAs contact protofilaments B10 and B9. **f** Cross-sectional view shows that the stalks of dyneins fα and fβ binding to protofilament B9. **g** Fitting of AlphaFold2 Multimer predicted model of CFAP57A/C (UniProtID Q234G8 and W7WWA2) into the cryo-EM map shows CFAP57A/C coiled coil region interacts with stem of dyneins d and g. **h, j** Part of CFAP57A/C coiled coil interacts with the base of the RS3. Colors: CFAP57A/C coiled coil, green; RS3, blue; dynein, light pink. Scale bar, 20 nm.

Supplementary Tables

Supplementary Table 1. The method of localizing and modelling of DRC components in cryo-EM maps.

DRC subunits	UniProt ID	Gene name	Region Coot Model	Region AF localizing	Localization rationales
DRC1<br><br>DRC2	Q229S1<br><br>Q24DJ0	TTHERM_01345750<br><br>TTHERM_00971830	291-734<br><br>277-480	292-826<br><br>278-576	• The C-terminal of DRC1 (residue 291-734) modelled using Coot based on C. reinhardtii structure [1].<br>• The predicted structure of the linker domain in DRC1 and DRC2 using AlphaFold2 Multimer, revealing that they form a coiled-coil protein. The linker domain of DRC1 folds back in a manner that accurately aligns with our map.<br>• The cross-link data showed links between DRC1 and DRC2, indicating dimerization.
DRC3	I7MG46	TTHERM_00316370	—	31-575	• The subtomogram average showed that DRC3 is missing in *drc3* mutant [2].<br>• The AlphaFold2 model of DRC3 shows a unique arrangement of the LRR and 3-helical bundle domains, which corresponds precisely to our cryo-EM map.<br>• Localization of DRC3 aligns with the positions of the N- and C-termini of DRC3 that were identified using strep-Au labels and cryo-electron tomography (cryo-ET) [3].
DRC4A<br><br>DRC4B	Q23YW7<br><br>I7LT80	TTHERM_00857910<br><br>TTHERM_00649240	293-464<br><br>292-472	1-294<br><br>1-293	• The baseplate domain is modelled using Coot and the side chains fitted well in our density map.<br>• DRC4A cross link with DRC4B, indicating heterodimer formation.<br>• Pull-down assay showed DRC4A and DRC4B interact with each other.<br>• AlphaFold2 multimer prediction revealed that the structure of DRC4A and DRC4B form a coiled-coil protein, which is consistent with our cryo-EM density. However, the structure of DRC4A-A and DRC4B-B do not match with our map.
DRC5	Q24C31	TTHERM_00697450	—	1-461	• The AlphaFold2 predicted model of DRC5 precisely matches with the LRR density in our map, which surrounds DRC1,2,4.<br>• The AlphaFold2 multimer prediction showed that DRC5 interacts with DRC1,2 at the expected location, as indicated by our cryo-EM maps.<br>• The C-terminus of DRC5 in the atomic model aligns with the location of the C-terminal strep-Au tag [4, 5].
DRC6C	Q24CL2	TTHERM_01220370	—	1-345	• DRC5 and DRC6 lost in *drc5* mutants [6-8].<br>• The region close to the top part of the linker domain is lost in subtomogram average map of *drc5* mutant [6].<br>• DRC6C showed a cross-link with DRC12 in the top part of the linker domain.<br>• The AlphaFold2 multimer prediction of DRC12 and DRC6 is consistent with our cryo-EM map and cross-link data.<br>• The DRC6 in localize in the top part of the linker domain and interacts with adjacent B-tubule which is consistent with our subtomogram average and previous studies.
DRC7	I7MLZ4	TTHERM_00473320	—	1-852	• DRC7 is missing in the central part of the N-DRC structure in *drc7* mutant [4].<br>• DRC7 cross-links with the linker domain of DRC9/10.<br>• The linker domain of DRC9/10 is missing in *drc7* mutant[4].<br>• DRC7 cross-links with DRC3.<br>• The AlphaFold2 multimer prediction showed DRC7 interact with DRC3 and DRC9/10.

DRC8<br>DRC11A	W7WX86<br>I7LWE3	TTHERM_001232262<br>TTHERM_00151820	—	1-185<br>1-862	• DRC8 is missing in *drc11* mutant [4].<br>• The subtomogram average showed that DRC11 density in the N-DRC proximal lobe is missing in *drc11* mutant [4].<br>• The AlphaFold2 multimer and Hdock prediction showed that two copies of DRC8 can interact with DRC11.<br>• Assembline localized DRC8 close to DRC11 in our cryo-EM density map, which is highly consistent with AlphaFold2 multimer and Hdock prediction.<br>• DRC11A cross-links with the N-terminal domain of DRC4B.
DRC9<br>DRC10	Q23S05<br>A4VD15	TTHERM_00625950<br>TTHERM_00535929	—	1-372<br>1-434	• Our cryo-EM map revealed an uncharacterized coiled-coil density which interacts with DRC3.<br>• AlphaFold2 multimer of DRC9/10 and DRC3 showed DRC3 interacts with DRC9/10 which is consistent with our map.<br>• AlphaFold2 multimer model of DRC9/10 is consistent with coiled-coil density in our map.<br>• The N-terminus of DRC9/10 precisely fits in the N-DRC bulb density.<br>• DRC9/10 cross-links with DRC7.
DRC12	Q22RH5	TTHERM_00016120	—	1-187	• Our cryo-EM map showed unidentified coiled-coil density which interacts with DRC1/2 and DRC6.<br>• DRC12 cross-links with DRC1 and DRC6.<br>• AlphaFold2 model of DRC12 matches with the coiled-coil density in our cryo-EM map.<br>• The positive charge of flexible loops in the top part of the linker domain of DRC12 is consistent with the proposed electrostatic interaction between the N-DRC and polyglutamylated tubulin [9].<br>• BioID result showed that DRC12 is biotinylated when DRC1-HA-BirA* and DRC2-HA-BirA* tagged as baits.<br>• The RNA expression of DRC12 from different human tissues correlates well with that of DRC1 and DRC2.
CCDC96<br>CCDC113<br>CFAP337	I7M6D6<br>Q22KK0<br>I7MM07	TTHERM_00529650<br>TTHERM_00312810<br>TTHERM_00475060	636-777<br>212-355	104-408,<br>442-626<br>1-202<br>36-865	• CCDC96 and CCDC113 interact with each other [10].<br>• Subtomogram average showed that CCDC96/113 are localized between the N-DRC and RS3 and IDAg.<br>• CCDC96 cross-links with CCDC113.<br>• CCDC96 cross-links with CFAP91.<br>• AlphaFold2 multimer prediction showed that CCDC96 and CCDC113 form a coiled-coil and fold back close to the N-terminal region, which is highly consistent with our cryo-EM map.<br>• The N-terminal domain of CCDC96 is not conserved and localize in top part of the N-DRC complex.<br>• CFAP337 cross-link with CCDC96.<br>• CFAP337 is missing in MS/MS of CCDC96-KO cells.<br>• The AlphaFold2 predicted structure of CFAP337 match with two WD domains in our map.<br>• CFAP337B (UniProtID I7MKT5), paralog of CFAP337A is biotinylated when DRC3-HA-BirA* tagged as baits.
CCDC39<br>CCDC40	Q23BW0<br>Q233L0	TTHERM_00227220<br>TTHERM_00391400	533-763<br>516-698	101-308,<br>530-310<br>53-257,<br>259-480	• CCDC39 cross-links with CCDC40 and DRC2.<br>• High resolution allowed us to model a short segment of the CCDC39/40 coiled coil using Coot and fit the side chains based on *C. reinhardtii* [1].<br>• CCDC40 cross-links with CFAP91 and DRC4B.
CFAP91	I7LWP7	TTHERM_00578560	169-347	—	• High resolution allowed us to model a short segment of the CFAP91 using Coot and fit the side chains based on *C. reinhardtii* [1].<br>• CFAP91 cross-links with CCDC96 and CCDC40.
CFAP20	Q22NU3	TTHERM_00418580	1-187	—	• High resolution allowed us to model complete CFAP20.

CFAP57A<br><br>CFAP57C	Q234G8<br><br>W7WWA2	TTHERM_00105300<br><br>TTHERM_00052490	—	1-883,<br>909-1147,<br>1161-1230<br>1-872,<br>898-1137,<br>1151-1209	• Cross-links between CFAP57A and CFAP57C indicate that they form a heterodimer.<br>• The AlphaFold2 multimer model of CFAP57A and CFAP57C forms coiled-coiled with the WD domain, which exactly match with the coiled-coiled and WD40 domain density in our cryo-EM map.<br>• The density of CFAP57 is found near CFAP337, which is consistent with previous study indicating the absence of CFAP337 in CFAP57-KO cells [11].<br>• CFAP57A was highly biotinylated in cells expressing CCDC96-HA-BirA* or CCDC113-HA-BirA* fusion proteins [10].
Calm 7-2	I7MDA9	TTHERM_00549500	—	1-202	• DRC6C cross-links with Calmodulin 7-2 protein.<br>• AlphaFold2 model of Calmodulin 7-2 matches with small globular densities in our map.<br>• AlphaFold2 multimer model of Calmodulin 7-2/DRC9/10 showed two copies of Calmodulin 7-2 interact with DRC9/10 which is consistent with our cryo-EM map.<br>• Previous study showed that CaM-like protein co-purified with N-DRC providing evidence for the presence of Calmodulin 7-2 in the N-DRC [5].

Supplementary Table 2. The homologs of N-DRC subunits of *T. thermophila* and human based on N-DRC subunits of *C. reinhardtii*. The emPAI score (no-salt treatment) and salt resistance were calculated from the mass spectrometry data of *T. thermophila* axonemes on DataDryad [12].

DRC subunits	*C. reinhardtii*	Human	*T. thermophila*	Resistant to salt	emPAI score
DRC1	P0DL09	Q96MC2	Q229S1	Yes	1.457
DRC2	A8JB22	Q8IXS2	Q24DJ0	Yes	3.253
DRC3	A8IVX2	Q9H069	I7MG46	Yes	3.685
DRC4A	Q7XJ96	O95995	Q23YW7	Yes	2.499
DRC4B	Q7XJ96	O95995	I7LT80	Yes	3.567
DRC5	A8HMZ4	Q5JU00	Q24C31	Yes	1.521
DRC6A	A8JHD7	Q8NEE6	I7M1R2	Yes	1.188
DRC6B	A8JHD7	Q8NEE6	I7MIQ9	No	0.202
DRC6C	A8JHD7	Q8NEE6	Q24CL2	Yes	1.991
DRC7	A0A2K3CXC4	Q8IY82	I7MLZ4	Yes	2.389
DRC8	A8J3A0	Q5VUJ9	W7WX86	Yes	1.823
DRC9	A0A2K3E6B1	Q9H095	Q23S05	Yes	2.442
DRC10	A8J0N6	Q96DY2	A4VD15	Yes	1.883
DRC11A	A0A2K3DJI6	Q86XH1	I7LWE3	Yes	2.326
DRC11B	A0A2K3DJI6	Q86XH1	W7XI81	No	0.316
DRC12	A0A2K3DW17	Q494R4	Q22RH5	Yes	2.401
CFAP337A	A0A2K3DIJ7	A0A804HJG6	I7MM07	No	1.694
CFAP337B	A0A2K3DIJ7	A0A804HJG6	I7MKT5	No	3.399
CCDC96	A0A2K3DWC0	Q2M329	I7M6D6	Yes	1.697
CCDC113	A0A2K3CQ22	Q9H0I3	Q22KK0	Yes	3.358
CCDC39	A0A0A1H1F6	Q9UFE4	Q23BW0	No	3.230
CCDC40	A0A0A1GYE8	Q4G0X9	Q233L0	No	2.933
CFAP91	A0A2K3DJU2	Q7Z4T9	I7LWP7	No	0.319
CFAP20	A8IU92	Q9Y6A4	Q22NU3	No	0.648
CFAP57A	A0A2K3DTF5	A0A087WVY5	Q234G8	No	1.225
CFAP57C	A0A2K3DV21	A0A087WVY5	W7WWA2	No	0.924
CFAP57B	A0A2K3DRH4	A0A087WVY5	Q23CU1	No	0.609
CFAP57D		A0A087WVY5	Q22N63	No	0.402
Calmodulin 7-2	P04352	P0DP25	I7MDA9	Yes	1.427

Supplementary Table 3. Intermolecular cross link data identified from N-DRC.

Protein 1	UniProtID	Residue number	Protein 2	UniProtID	Residue number
CCDC39	Q23BW0	660	CFAP91	I7LWP7	218
CCDC39	Q23BW0	613	DRC2	Q24DJ0	342
CFAP91	I7LWP7	343	CCDC96	I7M6D6	750
CFAP91	I7LWP7	347	CCDC96	I7M6D6	750
CFAP91	I7LWP7	343	CCDC40	Q233L0	542
CFAP91	I7LWP7	343	CCDC40	Q233L0	531
DRC4A	Q23YW7	123	DRC1	Q229S1	150
DRC12	Q22RH5	83	DRC1	Q229S1	138
DRC1	Q229S1	178	DRC4A	Q23YW7	149
DRC1	Q229S1	180	DRC2	Q24DJ0	172
DRC1	Q229S1	258	DRC2	Q24DJ0	247
CCDC96	I7M6D6	665	CCDC113	Q22KK0	245
CCDC96	I7M6D6	697	CCDC113	Q22KK0	274
CCDC96	I7M6D6	679	CCDC113	Q22KK0	259
CCDC113	Q22KK0	346	CCDC96	I7M6D6	761
CCDC113	Q22KK0	108	CCDC96	I7M6D6	532
CCDC113	Q22KK0	52	CCDC96	I7M6D6	532
CCDC113	Q22KK0	152	CCDC96	I7M6D6	573
DRC4B	I7LT80	332	CCDC40	Q233L0	542
DRC4B	I7LT80	322	DRC4A	Q23YW7	315
DRC4B	I7LT80	83	DRC4A	Q23YW7	73
DRC4B	I7LT80	201	DRC4A	Q23YW7	194
DRC4B	I7LT80	55	DRC11A	I7LWE3	551
DRC4B	I7LT80	56	DRC11A	I7LWE3	562
DRC4B	I7LT80	378	DRC2	Q24DJ0	367
DRC4B	I7LT80	261	DRC2	Q24DJ0	262
DRC4B	I7LT80	218	DRC2	Q24DJ0	255
DRC10	A4VD15	346	DRC7	I7MLZ4	250
DRC3	I7MG46	377	DRC7	I7MLZ4	566
DRC3	I7MG46	355	DRC7	I7MLZ4	221
CFAP91	I7LWP7	343	DRC4B	I7LT80	332
DRC2	Q24DJ0	228	DRC4B	I7LT80	233
CFAP337	I7MM07	1004	CCDC96	I7M6D6	533
CFAP57A	Q234G8	812	CFAP57C	W7WWA2	802
CFAP57A	Q234G8	1258	CFAP57C	W7WWA2	1266
DRC6C	Q24CL2	209	DRC12	Q22RH5	48
DRC6C	Q24CL2	222	Calmodulin 7-2	I7MDA9	63

Supplementary Table 4. Intramolecular cross-links identified from the N-DRC subunits.

Protein 1	UniProtID	Residue Number	Protein 2	UniProtID	Residue number
DRC4A	Q23YW7	100	DRC4A	Q23YW7	93
DRC5	Q24C31	397	DRC5	Q24C31	387
DRC5	Q24C31	91	DRC5	Q24C31	83
DRC1	Q229S1	187	DRC1	Q229S1	180
CCDC96	I7M6D6	351	CCDC96	I7M6D6	289
CCDC96	I7M6D6	368	CCDC96	I7M6D6	255
CCDC96	I7M6D6	380	CCDC96	I7M6D6	375
CCDC96	I7M6D6	351	CCDC96	I7M6D6	289
CCDC96	I7M6D6	255	CCDC96	I7M6D6	375
CCDC96	I7M6D6	255	CCDC96	I7M6D6	380
CCDC96	I7M6D6	263	CCDC96	I7M6D6	339
CCDC96	I7M6D6	380	CCDC96	I7M6D6	269
CCDC96	I7M6D6	255	CCDC96	I7M6D6	375
CCDC96	I7M6D6	263	CCDC96	I7M6D6	339
CCDC96	I7M6D6	351	CCDC96	I7M6D6	289
CCDC113	Q22KK0	108	CCDC113	Q22KK0	100
CCDC113	Q22KK0	52	CCDC113	Q22KK0	108
CCDC113	Q22KK0	280	CCDC113	Q22KK0	284
DRC11A	I7LWE3	309	DRC11A	I7LWE3	313
DRC11A	I7LWE3	559	DRC11A	I7LWE3	562
DRC11A	I7LWE3	831	DRC11A	I7LWE3	562
DRC7	I7MLZ4	488	DRC7	I7MLZ4	464
DRC7	I7MLZ4	433	DRC7	I7MLZ4	464
DRC7	I7MLZ4	576	DRC7	I7MLZ4	681
DRC7	I7MLZ4	576	DRC7	I7MLZ4	566
DRC10	A4VD15	277	DRC10	A4VD15	285
DRC10	A4VD15	239	DRC10	A4VD15	246
DRC3	I7MG46	386	DRC3	I7MG46	392
DRC3	I7MG46	463	DRC3	I7MG46	302
DRC3	I7MG46	53	DRC3	I7MG46	88
DRC3	I7MG46	119	DRC3	I7MG46	27
DRC3	I7MG46	385	DRC3	I7MG46	392

Supplementary Table 5. Mass spectrometry-based identification of ciliary proteins biotinylated in cells expressing the C-terminally -HA-BirA* tagged DRC proteins. Numbers X/Y: (X) number of all identified peptides (in a Mascot program, all significant matches), (Y) number of all unique peptide sequences (in a Mascot program, significant sequences).

		**Experiment 1**			**Experiment 2**				**Experiment 3**		
		**WT**	**DRC1-HA-BirA***	**DRC2-HA-BirA***	**WT**	**DRC1-HA-BirA***	**DRC2-HA-BirA***	**DRC3-HA-BirA***	**WT**	**DRC7-HA-BirA***	**DRC9-HA-BirA***
DRC1	TTHERM_01345750	1/1	31/20	16/14	0	9/7	10/7	0	0	0	0
DRC2	TTHERM_00971830	0	17/10	12/10	0	11/7	26/15	2/2	0	0	0
DRC3	TTHERM_00316370	0	18/10	17/11	0	3/2	6/4	17/11	0	0	0
DRC4A	TTHERM_00857910	2/2	23/14	20/11	0	10/9	21/13	2/2	0	1/1	0
DRC4B	TTHERM_00649240	3/1	23/12	23/13	0	5/4	10/8	2/2	0	1/1	2/1
DRC5	TTHERM_00697450	0	0	1/1	0	0	1/1	0	0	2/2	0
DRC6A	TTHERM_00339720	0	0	0	0	0	1/1	0	0	0	0
DRC6B	TTHERM_00522350	0	0	0	0	0	0	0	0	0	0
DRC6C	TTHERM_01220370	0	17/7	6/4	0	0	1/1	0	0	0	0
DRC7	TTHERM_00473320	0	19/11	16/14	0	3/2	3/3	1/1	0	43/25	0
DRC8	TTHERM_001232262	0	0	0	0	1/1	0	0	0	0	0
DRC9	TTHERM_00625950	6/4	12/10	16/8	0	1/1	7/6	3/3	0	0	7/4
DRC10	TTHERM_00535929	0	0	0	0	6/4	13/9	3/3	0	0	0
DRC11A	TTHERM_00151820	2/2	13/8	14/12	0	0	0	0	0	0	0
DRC11B	TTHERM_000268319	0	0	0	0	0	1/1	2/2	1/1	1/1	0
DRC12	TTHERM_00016120	0	0	0	0	3/2	1/1	0	0	0	0
CCDC96	TTHERM_00529650	1/1	29/18	8/6	0	1/1	0	0	0	0	0
CCDC113	TTHERM_00312810	4/4	27/16	17/11	0	45/20	1/1	1/1	0	0	0
CFAP337A	TTHERM_00475060	0	3/2	1/1	0	0	0	0	0	0	0
CFAP337B	TTHERM_00218510	0	1/1	0	0	0	0	2/2	0	0	0
CFAP91	TTHERM_00578560	0	4/3	6/4	0	0	0	0	0	0	0
CFAP57A	TTHERM_00105300	0	45/26	24/18	0	29/21	7/6	0	0	0	0
CFAP57B	TTHERM_00052490	0	4/3	2/2	0	0	0	0	0	0	0
CFAP57C	TTHERM_00214710	0	0	0	0	0	0	0	0	0	0
CFAP57D	TTHERM_000681920	0	0	0	0	32/23	13/9	0	0	0	0
CCDC39	TTHERM_00227220	0	2/2	2/2	0	0	0	0	0	0	0
CCDC40	TTHERM_00391400	0	3/2	0		0	0	0	0	0	0
CFAP20	TTHERM_00418580	11/7	25/7	25/8	0	1/1	2/1	0	0	0	0
Calmodulin 7-2	TTHERM_00549500	1/1	3/3	7/3	0	0	2/2	1/1	0	0	0

Supplementary Table 6. Normalized transcript per million (nTPM) RNA expression levels of DRCs in different human tissues obtained from The Human Protein Atlas on December 20, 2022

**Tissue**	**DRC1**	**DRC2**	**DRC3**	**DRC4**	**DRC5**	**DRC6**	**DRC7**	**DRC8**	**DRC9**	**DRC10**	**DRC11**	**DRC12**
Cerebillum	2.7	0.0	1.6	10.0	6.2	0.2	1.2	14.4	8.4	2.7	18.1	1.4
Choroid plexus	2.7	3.4	18.7	12.3	1.3	4.4	11.4	26.7	25.2	8.4	22.7	10.3
Fallopian Tube	11.1	26.6	61.4	18.9	14.4	5.0	21.1	36.1	52.8	22.0	11	39.3
Lung	1.5	2.9	5.3	7.1	1.4	0.5	1.9	12.6	9.1	2.6	2.7	3.1
Midbrain	1.9	2.3	11.4	10.1	3.3	2.2	9.3	16.9	19.8	5.7	27	7.3
Parathyroid gland	2.8	1.4	6.6	16.3	0.0	0.5	0.7	15.4	4.4	5.1	1.4	0.0
Pituitary gland	3.3	2.1	9.9	14.1	0.5	0.6	1.0	8.5	6.1	3.9	10.3	3.2
Testis	1.8	37.3	11.9	16.8	30	20.7	46.1	28	43.9	44.3	4.5	20.8

Supplementary Table 7. Primers used in this study.

Gene	Primer's name	Primers' nucleotide sequence
expression of C-terminally –HA-BirA* tagged DRC proteins under the control of respective native promoter		
DRC1	DRC1-Bir-cod-MluI-F	AAAT**ACGCGT**GAGGAAGCAAGAAAATAACTTCTTTT G
	DRC1-Bir-cod-BamHI-R	AATT**GGATCC**TCTTTGTCCTTAATCTATATTAATGAGTCTTG
	DRC1-Bir-3UTR-PstI-F	AAAT**CTGCAG**CCTACTCTAAAAAAATTATTCTCCTGATAAAA
	DRC1-Bir-3UTR-XhoI-R	AATT**CTCGAG**AATCTTCAAAAAGTAAGTAGATTGATAGATAG
DRC2	DRC2-Bir-cod-MluI-F	AAAT**ACGCGT**CATGCCCATAATAAGAATAGAAAGTAGGA
	DRC2-Bir-cod-BamHI-R	AATT**GGATCC**CTAGAATGGAGGAGCTCTTTATCC
	DRC2-Bir-3UTR-PstI-F	AAAT**CTGCAG**CACTCTATCCATTAATTACATTTGTATCTG
	DRC2-Bir-3UTR-XhoI-R	AATT**CTCGAG**CGAACTTTTGGTTGAGTTCAAAAG
DRC3	DRC3-Bir-cod-MluI-F	AATT**ACGCGT**GGGAGAAAGAAGCTGCTAATG
	DRC3-Bir-cod-BamHI-R	AATT**GGATCC**ATTTTCATGATCGGATTCATCATCTC
	DRC3-Bir-3UTR-PstI-F	AATT**CTGCAG**AGATCTATCTATCTATTGACTTAATTGATTTGT
	DRC3-Bir-3UTR-XhoI-R	AATT**CTCGAG**ATCGATCTCAATAATGTAGATTTACCGACGGA AGGG
DRC7	DRC7-Bir-cod-MluI-F	AATT**ACGCGT**CTCATTACCCTGCTAATGAATAGATTC
	DRC7-Bir-cod-BamHI-R	AATT**GGATCC**CTTTCTATGTAAGGCTGCTAAACG
	DRC7-Bir-3UTR-PstI-F	AATT**CTGCAG**CGCTAGATAAAAATATTAGATTGGGCA
	DRC7-Bir-3UTR-XhoI-R	AATT**CTCGAG**CTCAGATGATTTACAAATAACTCCTAGTG
DRC9	DRC9-Bir-cod-MluI-F	AATT**ACGCGT**AGATCTGTGTAGACTGTTTAAAGAAAATCCTGA
	DRC9-Bir-cod-BamHI-R	AATT**GGATCC**TTTCTTTTTCTTTTTGCCTTTCTTTCTC
	DRC9-Bir-3UTR-PstI-F	AATT**CTGCAG**GTTTGTACAATTTAAAATTAGTATTCAAACAAAGA
	DRC9-Bir-3UTR-XhoI-R	AATT**CTCGAG**AAGCTTGAAGTTAAAATCTTCTATCTCAAATTCCATG
Overexpression of DRC proteins under the control of MTT1 promoter		
DRC3	DRC3-oex-MluI-F	AATT**ACGCGT**TATGTCGAATTACATTAGCAACTACG
	DRC3-oex-BamHI-R	AATT**GGATCC**ATTTTCATGATCGGATTCATCATCTC
DRC4A	DRC4A-oex-MluI-F	AATT**ACGCGT**TATGCCTCCAAAAAAAGCTAAAGG
	DRC4A-oex-BamHI-R	AATT**GGATCC**AGAAGAGACTAAACCAGCAGG
DRC4B	DRC4B-oex-MluI-F	AATT**ACGCGT**TATGTCGAAAGCTGCTAAAGC
	DRC4B-oex-BamHI-R	AATT**GGATCC**ATCTGTGTTAGTAGGTACTAAAGGAT
DRC11B	DRC11B-oex-MluI-F	AATT**ACGCGT**TATGTCTACGAATTTTTATAATTTGCAATGGAAGCAG
	DRC11B-oex-BamHI-R	AATT**GGATCC**TTTCTTCTTTTTCTTAGCTTTACCACC

Supplementary Movies

Supplementary Movie 1: The molecular architecture of the N-DRC on the DMT.

References

1. Gui, M., et al., *Structures of radial spokes and associated complexes important for ciliary motility.* Nat Struct Mol Biol, 2021. **28**(1): p. 29-37.
2. Awata, J., et al., *DRC3 connects the N-DRC to dynein g to regulate flagellar waveform.* Mol Biol Cell, 2015. **26**(15): p. 2788-2800.
3. Song, K., et al., *In situ localization of N and C termini of subunits of the flagellar nexin-dynein regulatory complex (N-DRC) using SNAP tag and cryo-electron tomography.* J Biol Chem, 2015. **290**(9): p. 5341-53.
4. Gui, L., et al., *Scaffold subunits support associated subunit assembly in the Chlamydomonas ciliary nexin-dynein regulatory complex.* Proc Natl Acad Sci U S A, 2019. **116**(46): p. 23152-23162.
5. Oda, T., H. Yanagisawa, and M. Kikkawa, *Detailed structural and biochemical characterization of the nexin-dynein regulatory complex.* Mol Biol Cell, 2015. **26**(2): p. 294-304.
6. Heuser, T., et al., *The dynein regulatory complex is the nexin link and a major regulatory node in cilia and flagella.* J Cell Biol, 2009. **187**(6): p. 921-933.
7. Bower, R., et al., *The N-DRC forms a conserved biochemical complex that maintains outer doublet alignment and limits microtubule sliding in motile axonemes.* Mol Biol Cell, 2013. **24**(8): p. 1134-1152.
8. Lin, J., et al., *Building blocks of the nexin-dynein regulatory complex in Chlamydomonas flagella.* J Biol Chem, 2011. **286**(33): p. 29175-29191.
9. Kubo, T. and T. Oda, *Electrostatic interaction between polyglutamylated tubulin and the nexin-dynein regulatory complex regulates flagellar motility.* Mol Biol Cell, 2017. **28**(17): p. 2260-2266.
10. Bazan, R., et al., *Ccdc113/Ccdc96 complex, a novel regulator of ciliary beating that connects radial spoke 3 to dynein g and the nexin link.* PLoS Genet, 2021. **17**(3): p. e1009388.
11. Lin, J., et al., *FAP57/WDR65 targets assembly of a subset of inner arm dyneins and connects to regulatory hubs in cilia.* Mol Biol Cell, 2019. **30**(21): p. 2659-2680.
12. Bui, K.H., *Mass spectrometry of natively decorated doublet microtubule from Tetrahymena thermophila WT and mutants.* Dryad, 2021.
