# Supplementary material for: Integrated modeling of the Nexin-dynein regulatory complex reveals its regulatory mechanism
Source: Nat Commun. 2023 Sep 15;14:5741. doi: 10.1038/s41467-023-41480-7 (PMC10504270; doi:10.1038/s41467-023-41480-7)
Supplement: Supplementary file 3 — Description of Additional Supplementary Files [file 41467_2023_41480_MOESM3_ESM.pdf]

Description of Additional Supplementary Files

File Name: Supplementary Movie 1
Description: The molecular architecture of the N-DRC on the DMT.
