# Supplementary material for: Integrated modeling of the Nexin-dynein regulatory complex reveals its regulatory mechanism
Source: Nat Commun. 2023 Sep 15;14:5741. doi: 10.1038/s41467-023-41480-7 (PMC10504270; doi:10.1038/s41467-023-41480-7)
Supplement: Supplementary file 5 — Reporting Summary [file 41467_2023_41480_MOESM5_ESM.pdf]

nature portfolio

Corresponding author(s): Khanh Huy Bui, Dorota Wloga

Last updated by author(s): Aug 30, 2023

Reporting Summary

Nature Portfolio wishes to improve the reproducibility of the work that we publish. This form provides structure for consistency and transparency in reporting. For further information on Nature Portfolio policies, see our Editorial Policies and the Editorial Policy Checklist.

Statistics

For all statistical analyses, confirm that the following items are present in the figure legend, table legend, main text, or Methods section.

n/a	Confirmed	
☐	☒	The exact sample size (*n*) for each experimental group/condition, given as a discrete number and unit of measurement
☐	☒	A statement on whether measurements were taken from distinct samples or whether the same sample was measured repeatedly
☐	☒	The statistical test(s) used AND whether they are one- or two-sided<br>*Only common tests should be described solely by name; describe more complex techniques in the Methods section.*
☒	☐	A description of all covariates tested
☒	☐	A description of any assumptions or corrections, such as tests of normality and adjustment for multiple comparisons
☐	☒	A full description of the statistical parameters including central tendency (e.g. means) or other basic estimates (e.g. regression coefficient) AND variation (e.g. standard deviation) or associated estimates of uncertainty (e.g. confidence intervals)
☐	☒	For null hypothesis testing, the test statistic (e.g. F, t, r) with confidence intervals, effect sizes, degrees of freedom and P value noted<br>*Give P values as exact values whenever suitable.*
☒	☐	For Bayesian analysis, information on the choice of priors and Markov chain Monte Carlo settings
☒	☐	For hierarchical and complex designs, identification of the appropriate level for tests and full reporting of outcomes
☒	☐	Estimates of effect sizes (e.g. Cohen's *d*, Pearson's *r*), indicating how they were calculated

Our web collection on statistics for biologists contains articles on many of the points above.

Software and code

Policy information about availability of computer code

Data collection	SerialEM 4.0 (David N. Mastronarde, https://bio3d.colorado.edu/SerialEM/)
Data analysis	MotionCor2 V_1.4.0 (Zheng, S. et al. 2017. MotionCor2: anisotropic correction of beam-induced motion for improved cryo-electron microscopy. Nat Methods 14, 331–332. https://doi.org/10.1038/nmeth.4193)<br>Relion V_3.0 and V_3.1.2 (Zivanov, J. et al. 2018. New tools for automated high-resolution cryo-EM structure determination in RELION-3. eLife 7:e42166)<br>Relion V_4.0 (Zivanov, J. et al., 2022. A Bayesian approach to single-particle electron cryo-tomography in RELION-4.0. eLife, 11. doi:10.7554/eLife.83724)<br>Gctf V_1.06 (Zhang K. 2016. Gctf: Real-time CTF determination and correction. J Struct Biol, 193;1, 1–12. https://doi.org/10.1016/j.jsb.2015.11.003)<br>e2helixboxer EMAN2 V_2.31 (Tang, G. et al. 2007. EMAN2: an extensible image processing suite for electron microscopy. J Struct Biol, 157;1, 38–46. https://doi.org/10.1016/j.jsb.2006.05.009)<br>IMOD V_4.11 (Mastronarde, D. N. 2005. Automated electron microscope tomography using robust prediction of specimen movements. J Struct Biol, 152;1, 36-51. doi:10.1016/j.jsb.2005.07.007)<br>Alignframes V_1 (Mastronarde, D. N., & Held, S. R. 2017. Automated tilt series alignment and tomographic reconstruction in IMOD. J Struct Biol, 197;2, 102-113. doi:10.1016/j.jsb.2016.07.011)<br>Topaz V_0.2.4 (Bepler, T., Morin, A., Rapp, M. et al. 2019. Positive-unlabeled convolutional neural networks for particle picking in cryo-electron micrographs. Nat Methods 16, 1153–1160. https://doi.org/10.1038/s41592-019-0575-8)<br>Iterative Helical Real Space Reconstruction V_0 (Egelman E. H. 2007. The iterative helical real space reconstruction method: surmounting the problems posed by real polymers. J Struct Biol, 157;1, 83–94. https://doi.org/10.1016/j.jsb.2006.05.015)

Frealign V_9.11 (Grigorieff N. 2016. Frealign: An Exploratory Tool for Single-Particle Cryo-EM. Methods Enzymol, 579, 191–226. https://doi.org/10.1016/bs.mie.2016.04.013)
DeepEmhancer V_0.13 (Sanchez-Garcia, R. et al. 2021. DeepEMhancer: a deep learning solution for cryo-EM volume post-processing. Commun Biol 4, 874. https://doi.org/10.1038/s42003-021-02399-1)
Modeller V_10.3 (Webb, B., & Sali, A. 2016. Comparative Protein Structure Modeling Using MODELLER. Current protocols in bioinformatics, 54, 5.6.1–5.6.37. https://doi.org/10.1002/cpbi.3)
ColabFold V_0 (Mirdita, M. et al. 2022. ColabFold: making protein folding accessible to all. Nat Methods 19, 679–682. https://doi.org/10.1038/s41592-022-01488-1)
AlphaFold2 V_2.3.0 (Jumper, J. et al. 2021. Highly accurate protein structure prediction with AlphaFold. Nature, 596(7873), 583-589. doi:10.1038/s41586-021-03819-2)
UCSF ChimeraX V_1.4 (Goddard, T. D. et al. 2018. UCSF ChimeraX: Meeting modern challenges in visualization and analysis. Protein science 27;1, 14–25. https://doi.org/10.1002/pro.3235)
DeepTracer V_0 (Pfab, J. et al. 2021. "DeepTracer for fast de novo cryo-EM protein structure modeling and special studies on CoV-related complexes", PNAS, 118;2, e2017525118; DOI: 10.1073/pnas.2017525118.)
Coot V_0.9.8.3 (Emsley, P. et al. 2010. Features and development of Coot. Acta crystallographica. Section D, Biological crystallography, 66;Pt 4, 486–501. https://doi.org/10.1107/S0907444910007493)
WARP V_1.0.9 (Tegunov, D., Cramer, P. 2019. Real-time cryo-electron microscopy data preprocessing with Warp. Nat Methods 16, 1146–1152. https://doi.org/10.1038/s41592-019-0580-y)
Phenix 1.20.1-4487 (Liebschner, D. et al. 2019. Acta Cryst. D75, 861-877. https://doi.org/10.1107/S0907444909052925)
Pymol V_2.5.2 (Schrodinger, LLC)
Scaffold V_4.8.4 (Proteome Software Inc.)
Axoneme Align V_0 (Bui, K. H., & Ishikawa, T. 2013. 3D structural analysis of flagella/cilia by cryo-electron tomography. Methods Enzymol, 524, 305-323. doi:10.1016/B978-0-12-397945-2.00017-2)
Assembline V_1.0 (Rantos, V. et al. 2022. Integrative structural modeling of macromolecular complexes using Assembline. Nat Protoc 17, 152–176. https://doi.org/10.1038/s41596-021-00640-z)

For manuscripts utilizing custom algorithms or software that are central to the research but not yet described in published literature, software must be made available to editors and reviewers. We strongly encourage code deposition in a community repository (e.g. GitHub). See the Nature Portfolio guidelines for submitting code & software for further information.

Data

Policy information about availability of data

All manuscripts must include a data availability statement. This statement should provide the following information, where applicable:

- Accession codes, unique identifiers, or web links for publicly available datasets
- A description of any restrictions on data availability
- For clinical datasets or third party data, please ensure that the statement adheres to our policy

All data produced and/or used in this study are available in the following databases:

Cryo-EM maps of combined 96-nm Tetrahymena doublet, combined N-DRC base plate part Tetrahymena are in the Electron Microscopy Data Bank database with the following EMDB IDs: EMD-41284, EMD-41189, EMD-41251.
Cryo-EM maps of focused refinement of the N-DRC from the Tetrahymena WT subtomogram average DMT are in the Electron Microscopy Data Bank database with the following EMDB IDs: EMD-41270.
Model coordinates for the baseplate, Linker, and N-DRC and associated proteins are in the RCSB Protein Data Bank database with the following PDB IDs: 8TEK, 8TH8, and 8TID

Human research participants

Policy information about studies involving human research participants and Sex and Gender in Research.

Reporting on sex and gender	this information has not been collected
Population characteristics	n/a
Recruitment	n/a
Ethics oversight	n/a

Note that full information on the approval of the study protocol must also be provided in the manuscript.

Field-specific reporting

Please select the one below that is the best fit for your research. If you are not sure, read the appropriate sections before making your selection.

- [x] Life sciences
- [ ] Behavioural & social sciences
- [ ] Ecological, evolutionary & environmental sciences

For a reference copy of the document with all sections, see nature.com/documents/nr-reporting-summary-flat.pdf

Life sciences study design

All studies must disclose on these points even when the disclosure is negative.

Sample size	There were no statistical methods used to predetermine sample size. For BioID experiments, we included two or more independent biological replicates prepared under the same conditions. For each biological replicate, we used the same strain grown from separate Tetrahymena bean stocks. For each biological replicate, we grew the strains in freshly prepared media to very similar densities for small scale growth, large scale growth, as well as the density at harvest. For each biological replicate, fresh buffers were prepared. For all cryoEM experiments, each map was obtained from data acquisition of tens (cryoET) to thousands (cryoEM) of movies of ciliary sample. For all datasets, the ciliary sample was purified from approximately 10 L of Tetrahymena culture.
Data exclusions	No data were excluded from analysis.
Replication	For our cryoEM experiments, our two half-maps generated from randomly selected data were in agreement.
Randomization	Randomization was not performed because the cryoEM, MS, and genetics analyses was done according to strain/condition, and also it is not typical for those aforementioned studies.
Blinding	Blinding is not applicable to cryoEM, MS, or genetics analyses as performed in this manuscript. Our data are entirely based on quantitative analysis and are not subjective. Furthermore, there was no blinding in this study because it was not a clinical trial and there were no drugs or animal/human subjects. Our data analyses include equivalent measures to blinding: we ensure all of our findings are representative of randomly selected subsets of the entire dataset.

Reporting for specific materials, systems and methods

We require information from authors about some types of materials, experimental systems and methods used in many studies. Here, indicate whether each material, system or method listed is relevant to your study. If you are not sure if a list item applies to your research, read the appropriate section before selecting a response.

Materials & experimental systems

n/a	Involved in the study	
☐	☒	Antibodies
☐	☒	Eukaryotic cell lines
☒	☐	Palaeontology and archaeology
☒	☐	Animals and other organisms
☒	☐	Clinical data
☒	☐	Dual use research of concern

Methods

n/a	Involved in the study	
☒	☐	ChIP-seq
☒	☐	Flow cytometry
☒	☐	MRI-based neuroimaging

Antibodies

Antibodies used	Anti-GFP antibody Rabbit, catalog number #ab6556 (Abcam)<br>Purified anti-HA.11 Epitope Tag Antibody (Previously Covance catalog# MMS-101P), clone 16B12, catalog number #901503 (BioLegend)<br>Polyclonal Peroxidase AffiniPure Goat Anti-Mouse IgG (H+L), catalog number #115-035-146, (Jackson ImmunoResearch Europe Ltd)<br>Goat Anti-Rabbit IgG, H & L Chain Specific Peroxidase Conjugate, catalog number #401315, (EMD Millipore)
Validation	All commercial antibodies were validated by the supplier. All lab-generated antibodies have been thoroughly tested and are commonly used in Western Blot.<br>Anti-GFP antibody Rabbit, catalog number #ab6556 (Abcam)(https://www.abcam.com/products/primary-antibodies/gfp-antibody-ab6556.html)<br>Purified anti-HA.11 Epitope Tag Antibody (Previously Covance catalog# MMS-101P), clone 16B12, catalog number #901503 (BioLegend)(https://www.biolegend.com/en-us/products/purified-anti-ha-11-epitope-tag-antibody-11374)<br>Polyclonal Peroxidase AffiniPure Goat Anti-Mouse IgG (H+L), catalog number #115-035-146, (Jackson ImmunoResearch Europe Ltd)(https://www.jacksonimmuno.com/catalog/products/115-035-146)<br>Goat Anti-Rabbit IgG, H & L Chain Specific Peroxidase Conjugate, catalog number #401315, (EMD Millipore)(https://www.emdmillipore.com/CA/en/product/Goat-Anti-Rabbit-IgG-H-L-Chain-Specific-Peroxidase-Conjugate,EMD_BIO-401315)

Eukaryotic cell lines

Policy information about cell lines and Sex and Gender in Research

Cell line source(s)	The wildtype CU428 Tetrahymena strains were obtained from the Tetrahymena Stock Center (Cornell University, Ithaca, NY,

	USA).
Authentication	The wildtype CU428 Tetrahymena strains were authenticated by the Tetrahymena Stock Center (Cornell University, Ithaca, NY, USA). The DRC3-HA, DRC4B-HA, DRC4A-HA, DRC11B-HA, and DRC11A-HA, DRC1-BirA, DRC2-BirA, DRC3-BirA, DRC7-BirA, DRC9-BirA strains were authenticated by the Wloga lab using a variety of genetic and biochemical screens. Constructs used to engineer Tetrahymena DRC mutants were obtained in the laboratory by cloning fragments of the genomic DNA amplified by PCR with addition of appropriate restriction enzymes. Proper cloning was confirmed by the restriction analyses and if required by DNA fragment sequencing. Constructs were introduced to the Tetrahymena cells by biolistic transformation and positive transformants were selected based on the resistance to paromomycin. The expression of the proteins were confirmed by immunofluorescence and western blot to compare the molecular weight of the expressed proteins with the theoretical molecular weight. Three to six independent clones was analyzed for each DRC mutant. (https://www.nencki.edu.pl/laboratories/laboratory-of-cytoskeleton-and-cilia-biology/)
Mycoplasma contamination	Not in Tetrahymena cells
Commonly misidentified lines (See ICLAC register)	No commonly misidentified lines were used in this study.
